# Making the most of an abundance of data

**DOI:** 10.1107/S205979832401204X

**Published:** 2025-01-01

**Authors:** Christoph Mueller-Dieckmann, Anna J. Warren, David G. Waterman

**Affiliations:** a Structural Biology Group European Synchrotron Radiation Facility (ESRF) 71 Avenue des Martyrs 38000Grenoble France; b Diamond Light Source Harwell Science and Innovation Campus, Fermi Avenue DidcotOX11 0DE United Kingdom; c Research Complex at Harwell UKRI–STFC Rutherford Appleton Laboratory Harwell DidcotOX11 0FA United Kingdom

**Keywords:** CCP4 Study Weekend, data

## Abstract

The Guest Editors introduce the special issue based on talks at the CCP4 Study Weekend 2023. The virtual issue is available at https://journals.iucr.org/special_issues/2024/CCP42023/.

The 2023 CCP4 Study Weekend was held as a hybrid event between the 4th and 6th of January at the East Midlands Conference Centre in Nottingham. The theme was ‘data’, but we organisers wished to broaden the scope beyond that of a typical Study Weekend devoted to crystallographic data. We chose the subtitle ‘from subtle details to big insights’ and included presentations about getting the most from structural biology data at all stages, from sample preparation to biophysical characterization, and on to structure solution. There was an atmosphere of reinvention and resurgence to the meeting, which we may put down to both discussions about the future of structural biology and the welcome return to in-person meetings following two years of virtual Study Weekends during the SARS-CoV-2 pandemic. Despite some trepidation about train strikes and our inexperience in handling hybrid meetings, the event was successful, and it is our pleasure to present this Special Issue of articles proceeding from the event.

We began the meeting on the evening of the 4th January, immediately following the Diamond MX User Meeting, with a session on integrative structural biology. Montserrat Soler-Lopez (ESRF, France) delivered an inspiring keynote lecture, taking Alzheimer’s disease as a showcase to explain how data from different experimental and theoretical techniques can be combined to aid increasing our understanding on the origin and development of the disease. This was followed by a panel discussion – a novel experiment for a Study Weekend programme – with the topic of structural biology data sources. Randy Read (University of Cambridge, UK) moderated the panel of experts: Sameer Velankar (EMBL–EBI, UK), Jim Naismith (RFI, UK), Annalisa Pastore (ESRF, France), Loes Kroon-Batenburg (Utrecht University, Netherlands), Kristina Djinovic-Carugo (EMBL Grenoble, France), Dave Stuart (Diamond Light Source, UK) and Gerard Bricogne (Global Phasing, UK) (see Fig. 1 ▸). Right from the start, the discussion did not shy away from the question on everyone’s minds, namely what do experiments bring us now that we can obtain better than anticipated predictions from *AlphaFold*? The answer is to keep improving experiments and to archive comprehensive data from them so that greater detail can be extracted as methods improve. Raw data deposition is best practice, and an appeal was made to the crystallographic community to do this as a matter of course. The cryo-EM community leads the way here and shows us that it is feasible. At the very least, the deposition of unmerged data would open new opportunities for structural analysis. The role of the PDB was clarified as the resource that gives a scientist’s eye view of experiments, linking to the relevant data sources and providing the tools for biological interpretation. Invigorated by the discussion, the day then closed with a poster session, giving students the opportunity to present their research to the wider meeting attendees. This was the first time that a poster session had been included in the Study Weekend and it was a popular addition to the meeting.

On the second morning, following the traditional What’s New in CCP4 session, the meeting continued with a session on the fundamentals of crystallographic data. Graeme Winter (Diamond Light Source, UK) set the scene by posing the question, what is data? This was followed by an educational introduction by Greta Assmann (PSI, Switzerland) to the statistics we use to judge and compare data sets. Kevin Dalton (Harvard University, USA) took us from this foundation right up to the cutting edge of statistical methods, with particular reference made to the *Careless* software and the novel ideas therein. The session was closed by Richard Gildea (Diamond Light Source, UK), who addressed the challenges inherent in handling multi-crystal data.

The first afternoon session addressed fundamentals related to samples and the experiment, beginning with an introduction by Ralf Flaig (Diamond Light Source, UK) about crystallo­graphic data-collection strategies and processing pipelines for the 21st century. Despite our best efforts to collect optimal data, radiation damage remains a serious issue, and Kathryn Shelley (University of Washington, USA) showed us how to quantify specific radiation damage in protein crystal structures, while also advising on how to both detect and avoid it where possible. The session continued with practical advice from Maria Garcia Alai (EMBL Hamburg, Germany) on how to prepare good samples for a variety of molecular biophysics techniques. Finally, Phillippe Carpentier (CNRS IRIG-LCBM Grenoble, France) described how to enhance information from diffraction data using high-pressure experiments that can introduce gas molecules into protein structures, for purposes such as tracing channels, inducing structural modifications and flash-cooling without cryoprotection.

The late afternoon session moved on to the topic of choosing your source, in recognition that we have more options than just sending crystals to synchrotron beamlines. Meytal Landau (Israel Institute of Technology, Israel) began with an inspiring lecture about the unpredictable structures of virulence factors and antimicrobial amyloid fibrils. A combination of high-resolution crystallographic structures of peptide fragments using microfocus beams, and cryo-EM structures of the supramolecular assemblies, gives structural insights across this wide family of tricky fibrillar proteins. Antoine Royant (IBS, France) moved on to introduce the increasingly important topic of time-resolved crystallography, and its range of applicability from femtoseconds to minutes at both XFEL and synchrotron sources. Arnaud Basle (Newcastle University, UK) then presented a counterpoint to the cutting-edge experiments performed at big facilities by reminding us of the capabilities of a modern ‘home’ source, which goes beyond testing and optimizing samples, even allowing in-house S-SAD structure solutions and drug-discovery campaigns. The final talk of the day from Hongyi Xu (Stockholm University, Sweden) presented protein crystallo­graphy in an electron microscope, describing how the 3DED, or MicroED, method can be used to solve the structures of biological macromolecules from nanocrystalline samples by electron diffraction.

The final day of the Study Weekend began with a session on ‘big data’. Derek Mendez (SLAC, USA) introduced the ExaFEL project, in which exascale computing is applied to crystallographic data processing. Learning how to use the fastest supercomputers is essential to provide rapid feedback for serial femtosecond crystallography. This requires an understanding of how to efficiently get data onto the compute nodes and how to adapt algorithms for their architecture. The second talk of the session came from Marjan Hadian-Jazi (La Trobe University, Australia), who presented the concept of robust statistics and how they apply to the big data sets obtained from serial crystallography. The problem of ‘data wrangling’ for time-resolved crystallography was addressed by Briony Yorke (University of Bradford, UK). Keeping track of many data sets and their relationships to one another can become a serious bookkeeping issue, but the method of Hadamard time-resolved crystallography provides an efficient framework for pump–probe experiments. For the final talk of the session, Kyle Morris (Diamond Light Source, UK) described a tool that is gathering data from all of microscope cryo-EM sessions at eBIC and providing ‘big data’ insights into how microscope and experimental configurations may influence data quality.

For the second morning session we moved on from challenges of data size to those of complexity under the title ‘between the Bragg spots’. Gloria Borgstahl (Nebraska Medical Centre, USA) began with a lecture on aperiodic crystal structures, including quasi-crystals as well as modulated crystals, and described the procedures necessary to get more from their data and even solve their structures. Clemens Vonrhein (Global Phasing, UK) then presented a number of cases where the availability of unmerged diffraction data was necessary to validate data-quality statistics, analyse anisotropy and improve structures in the case of radiation damage. Andrey Lebedev (UKRI–STFC, UK) warned us about the ‘things you do not want to see in your data’, presenting various real examples of diffraction data pathologies caused by partial crystal disorder, and how they can be dealt with. Steve Meisburger (CHESS, USA) then closed the session by considering the information contained in the diffuse scattering outside of the Bragg reflections, which tells us about the dynamics of a protein structure, ultimately informing us further on the protein function.

The final session of the meeting took a forward-looking stance surveying a new era in structural biology, in which we are embracing the power of AI tools. Dan Rigden (University of Liverpool, UK) began with a report on the 15th iteration of the CASP competition. Two years after the introduction of *AlphaFold*2, Dan described a dynamic field in which optimizations enhance *AlphaFold*2 predictions, rival methods such as language models show promise, and we heard hints of new breakthroughs to come. These developments are a boon to structural biologists and are highly accessible through resources such as the AlphaFold Database and new ESM Metagenomic Atlas. Sylvain Engilberge (IBS, France) highlighted that, despite the advancements in structure prediction, crystallography remains indispensable for understanding protein dynamics through various time-resolved techniques. He specifically emphasized the importance of integrating crystallography with complementary techniques such as *in crystallo* optical spectroscopy. This should provide crucial insights enhancing the interpretation of X-ray diffraction data, allowing a better understanding of protein function across time scales ranging from microseconds to minutes. Isabel Uson (CSIC, Barcelona) returned to the topic of structure prediction, but from a different angle, describing how guiding *AlphaFold* predictions can explore the conformational space of protein structures, providing design for experiments or valuable context for the interpretation of experimental results. Finally, Anastassis Perrakis (Netherlands Cancer Institute) closed the meeting with thoughts on the practical impact of protein structure-prediction methods, followed by a description of *AlphaFill*, a method to enhance the results of prediction by adding metals, ligands and cofactors.

We are very grateful to the behind-the-scenes staff, especially Karen McIntyre, Georgia Lomas, Emma Phillips, Jonathan Oldfield and Stuart Eyres, for holding everything together and dealing promptly with the few issues that did arise during the meeting. We would also like to thank Sam Horrell for organizing social activities, which is no mean feat for a hybrid meeting. The board-games evening was very well received by the in-person participants, while Slack channels such as ‘crafty crystallographers’ had a lot of activity from those joining virtually.

## Figures and Tables

**Figure 1:**
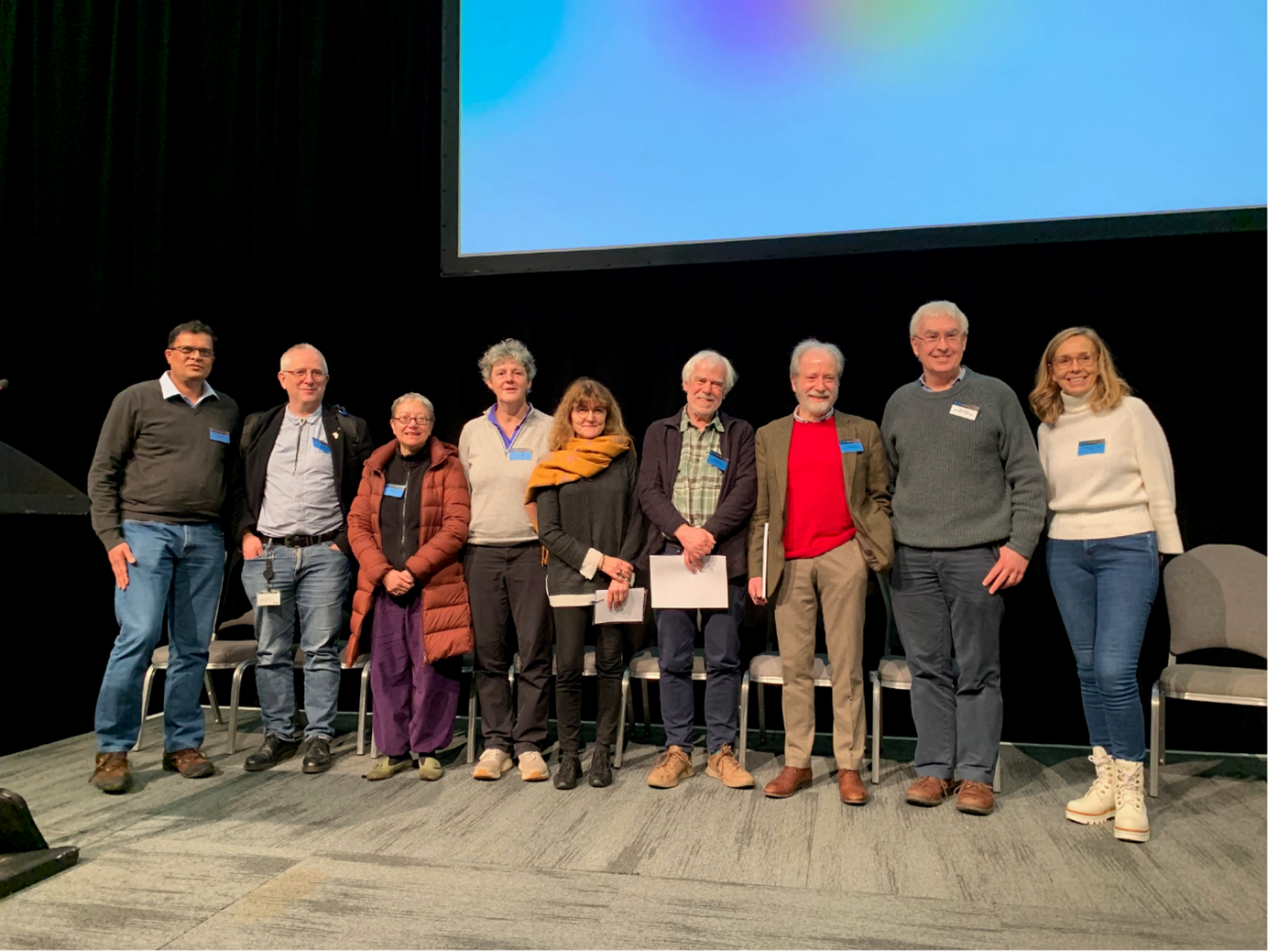
The integrative structural biology session speakers. From left to right: the panellists, Sameer Velankar, Jim Naismith, Annalisa Pastore, Loes Kroon-Batenburg, Kristina Djinovic-Carugo, Dave Stuart and Gerard Bricogne, joined by the panel moderator Randy Read and keynote speaker Montserrat Soler-Lopez.

